# Our Colaborers

**Published:** 1877-03-20

**Authors:** 


					﻿Editorial and Miscellaneous.
OUR COLABORERS.
In curtailing our list of co-laborers
the present year, no invidious distinction
is meant. We have placed those only
on the list who either by contributions
the last year, or otherwise, have indica-
ted a willingness to act for us. Our co-
laborers are expected to furnish us with
one or more short practical articles from
their own pen, to give us their aid and
influence in extending our circulation,
and to furnish us any useful and impor-
tant facts which may come under their
observation calculated to benefit the
profession, or interest our readers.
				

